# Glucose dysregulation and response to common anti-diabetic agents in the FATZO/Pco mouse

**DOI:** 10.1371/journal.pone.0179856

**Published:** 2017-06-22

**Authors:** Richard G. Peterson, Charles Van Jackson, Karen M. Zimmerman, Jorge Alsina-Fernandez, M. Dodson Michael, Paul J. Emmerson, Tamer Coskun

**Affiliations:** 1 Crown Bioscience - Indiana, Indianapolis, Indiana, United States of America; 2 Eli Lilly and Company, Indianapolis, Indiana, United States of America; East Tennessee State University, UNITED STATES

## Abstract

The FATZO/Pco mouse is the result of a cross of the C57BL/6J and AKR/J strains. The crossing of these two strains and the selective inbreeding for obesity, insulin resistance and hyperglycemia has resulted in an inbred strain exhibiting obesity in the presumed presence of an intact leptin pathway. Routinely used rodent models for obesity and diabetes research have a monogenic defect in leptin signaling that initiates obesity. Given that obesity and its sequelae in humans are polygenic in nature and not associated with leptin signaling defects, the FATZO mouse may represent a more translatable rodent model for study of obesity and its associated metabolic disturbances. The FATZO mouse develops obesity spontaneously when fed a normal chow diet. Glucose intolerance with increased insulin levels are apparent in FATZO mice as young as 6 weeks of age. These progress to hyperglycemia/pre-diabetes and frank diabetes with decreasing insulin levels as they age. The disease in these mice is multi-faceted, similar to the metabolic syndrome apparent in obese individuals, and thus provides a long pre-diabetic state for determining the preventive value of new interventions. We have assessed the utility of this new model for the pre-clinical screening of agents to stop or slow progression of the metabolic syndrome to severe diabetes. Our assessment included: 1) characterization of the spontaneous development of disease, 2) comparison of metabolic disturbances of FATZO mice to control mice and 3) validation of the model with regard to the effectiveness of current and emerging anti-diabetic agents; rosiglitazone, metformin and semaglutide. Conclusion: Male FATZO mice spontaneously develop significant metabolic disease when compared to normal controls while maintaining hyperglycemia in the presence of high leptin levels and hyperinsulinemia. The disease condition responds to commonly used antidiabetic agents.

## Introduction

The frequent and statistically significant clustering of adverse health conditions including obesity, insulin resistance, glucose intolerance, hypertension and dyslipidemia seen in many pre-diabetic individuals has led some clinicians to designate the cluster as metabolic syndrome [1–3]. A National Health Statistics Report in 2009 estimated that about 34% of adults over 20 years of age had metabolic syndrome at that time; with different levels of occurrence depending on sex, age and ethnicity [4]. The syndrome has been closely associated with cardiovascular disease and diabetes [5,6]. More recent data show that there has been an alarming increase in the syndrome in young adolescents, and, while this appears to have leveled off in children under 11, it is still increasing in adolescents 12–19 years of age [7]. The condition is multi-faceted with abnormalities in glucose and lipid homeostasis, which associates the disease with multiple co-morbidities such as obesity and multi-organ dysfunction (i.e., heart, vasculature, kidney, etc.) [8]. Metabolic syndrome has also been closely associated with a pro-inflammatory and pro-fibrotic state. Significant research is ongoing to establish the contribution of inflammation and fibrotic processes to multi-organ failure. It is generally agreed that a patient presenting with at least 3 of the following: hypertension, hyperglycemia, dyslipidemia, visceral adiposity, insulin resistance can be defined as having metabolic syndrome.

Current interventions for insulin resistance and diabetes, focus on four mechanisms: 1) enhancements of insulin secretion, 2) reduction of hepatic glucose production, 3) delay of carbohydrate absorption and 4) improvement in insulin sensitivity. These therapies are designed to improve hyperglycemia and insulin resistance, which are thought to be the hallmarks of the disease and ultimately lead to frank diabetes/organ failure. Unfortunately, the animal models currently available for pre-clinical screening of potential drug candidates do not accurately mimic the multi-faceted human condition [9]. Therefore, we have developed a new mouse strain that is anticipated to add significant predictive capabilities to pre-clinical efficacy studies for new therapies particularly as it relates to obesity, glucose intolerance, insulin resistance and hyperglycemia.

In general, the two mouse models (*ob/ob* and *db/db*) most commonly implemented in pre-clinical studies of type 2 diabetes/metabolic disturbances are characterized by monogenic mutations imparting dysfunction in the leptin signaling pathway [9–14]. While these models represent the major metabolic disturbances common to the syndrome, similar dysfunctions in the leptin pathway are very rare in humans [15–20]. In addition to these models, the diet-induced obese mouse (DIO) mouse model produced through prolonged feeding of a western diet is currently thought to most accurately reflect the pre-diabetic state due to induction of insulin resistance [9,11,21]. Many clinical studies have implicated the high carbohydrate/high fat diet as a causative factor in the increase of metabolic syndrome in humans.

The most commonly used method for establishing diet-induced obesity in mice is the feeding of a high fat diet to non-obese, non-diabetic C57BL/6 mice for a prolonged 8–16 week period. The resultant model exhibits marked obesity, hyperinsulinemia, insulin resistance and glucose intolerance. DIO mice develop peripheral leptin resistance primarily due to obesity [22–24], with no genetic functional defect in leptin or its receptor. In addition, the model appears to display a pro-inflammatory and pro-fibrotic state that is associated with the human metabolic syndrome [25,26]. The model has been published extensively in the evaluation of anti-diabetic and anti-obesity agents [27–31].

The FATZO/Pco mouse was produced by crossing of the AKR/J and C57BL/6J strains followed by selective inbreeding. These two strains are highly sensitive to the development of obesity, glucose intolerance and insulin resistance when fed high fat diets. Both strains develop leptin resistance while gaining adiposity [22–24,32]. However, under the same feeding conditions, AKR/J mice gain more weight, have higher carcass lipid content, plasma leptin, insulin and triglyceride concentrations, and are more insulin resistant but less hyperglycemic and glucose intolerant than C57BL/6J mice [33–35]. These phenotypic differences suggest that there is a difference in the genetic factors that cause diet-induced obesity and diabetes in these two strains. It should be noted that there is a 4-fold difference in adiposity even within the C57BL/6J strain when obesity is induced with a high fat diet [36]. The crossing of these two strains and the selective inbreeding of the subsequent generations has resulted in a strain exhibiting obesity in a pre-diabetic state, which slowly progresses to severe diabetes. This strain is valuable in studying the continuum of metabolic disturbances that accompany the conditions that lead to severe diabetes. The major advantages of the FATZO model are that it does not have the common obesity-producing monogenic (leptin) mutations, does not require high fat feeding to become obese and appears to develop a multi-faceted metabolic disease (obesity, hypertriglyceridemia, glucose intolerance, insulin resistance and hyperglycemia) very similar to the human disease.

In this study, we have characterized the spontaneous development of glucose dysregulation and the response to common anti-diabetic treatments (rosiglitazone, metformin and semaglutide) in the new FATZO mouse model.

## Methods

### Age related changes in glucose homeostasis

All animal studies were approved by Eli Lilly and Company’s and PreClinOmics’ Animal Care and Use Committees. FATZO/Pco mice were bred and maintained at PreClinOmics (now Crown Bioscience—Indiana). Male mice (n = 72) were housed 2 per cage. Room temperature was monitored and maintained at 72–77°F with the light cycle set at 12 hours (0600–1800 hr). Purina 5008 standard rodent chow and house water were provided *ad libitum*. Body weight, fed blood glucose and insulin were recorded at 2-week intervals from 6–22 weeks of age within two hours of the room lights coming on. Serum glucose was analyzed in fresh plasma using AU480 clinical analyzer (Beckman-Coulter, Brea, CA, USA). Insulin content in plasma was determined from frozen sample using a mouse/rat insulin kit (Meso Scale Discovery K152BZC-3, Rockville, MD, USA).

### Comparison to age matched controls

In a parallel study, body composition and glucose disposal in male FATZO mice (n = 6) were evaluated and compared to age matched control C57BL/6J mice (n = 6); Purina 5008 was the diet for both groups. Oral glucose tolerance tests (OGTT) were performed every 4 weeks from 6–18 weeks of age. Following a 12 hour fast, glucose (2 g/kg) was administered orally by gavage. Blood samples were taken via tail clip and glucose analyzed by StatStrip (Xpress, Data Science International, MN, USA) at 0, 30, 60, 90 and 120 minutes post-glucose load. The area of the blood glucose response curve corresponding to each animal was calculated by the trapezoid method [37], using each individual baseline blood glucose measurement prior to glucose administration as reference (t = 0). The sum of the trapezoidal areas between the 0, 30, 60, 90 and 120-minute time points corresponding to each animal was calculated to obtain the area under the curve (AUC). Serum triglycerides were assayed in the fed state in 6, 10 and 14 week old animals.

Body composition was assessed in conscious mice every 4 weeks from 6–18 weeks of age using qNMR (EchoMRI-700, Houston, TX, USA). Whole body qNMR [38] was performed just prior to initiation of fasting for OGTT. All data are presented as Mean ± SEM (n = 6/group).

### Response to metformin and rosiglitazone

Male FATZO mice (9 weeks of age, n = 40) were maintained on Purina 5008 regular rodent chow and reverse osmosis water *ad libitum*. Mice were housed 3 per cage and acclimated to study environment for 7 days prior to study. At 10 weeks of age, mice were fasted for 6 hours, body weight was recorded and an OGTT performed. Glucose and insulin were assayed at 0, 30, 60, 90, 120 and 180 minutes following a 2 g/kg glucose load. Glucose concentrations were obtained from StatStrip glucometer and insulin was assayed at each time-point using the insulin kit mentioned above. Insulin sensitivity index (ISI) was calculated using a formula modified from Matsuda and DeFronzo [39] by changing the numerator to 10,000 and using glucose and insulin AUCs instead of average glucose and insulin levels (100,000/square root of [fasting glucose x fasting insulin] x [glucose AUC x insulin AUC during OGTT]).

Animals were randomized into 3 groups of 10 based on baseline ISI and body weight. Groups were assigned to receive either vehicle (0.5% CMC, 0.1% Tween 80), rosiglitazone (KEMPROTEC Limited, U.K.) (10 mg/kg/day) or metformin (Toronto Research Chemicals, North York, Ontario, Canada) (150 mg/kg/day). Compounds were administered orally once daily by gavage for 8 weeks. Body weight was recorded weekly.

Following 8 weeks of treatment, OGTT was repeated one hour after compound administration. Whole blood was taken from tail clip and processed to serum. Treatment effects on glucose disposal, fed serum glucose, HbA1c, and ISI were compared to vehicle.

### Response to GLP-1 agonist semaglutide

Male FATZO mice (n = 32) were housed one per cage and maintained at constant room temperature (77–78°F) and fed Purina 5008 regular rodent chow from weaning until 12 weeks of age. At this time, a reversed 12-hour light cycle (to accommodate for glucose and OGTT activities) was initiated (dark cycle 0700–1900 hr) and the diet was changed to Purina 5015 for the remainder of the study. Animals were acclimated to this environment for 2–3 weeks. During the last 5 days of acclimation, animals were acclimated to handling by daily SQ administration of phosphate buffered saline (PBS). Twenty-four hours prior to study start, baseline values (whole blood glucose and body weight) were obtained 2–3 hours into the dark cycle. Blood sample was obtained in the fed state by tail clip for whole blood glucose level (StatStrip). Animals with body weight ≥ 40.0g and fed glucose level of ≥ 250 mg/dL were accepted for study, randomized to 4 groups of 8 based on body weight and fed glucose and assigned to receive either vehicle (20 mM citrate buffer, pH 7), or semaglutide 1.0, 3.0 or 10.0 nmol/kg, SQ, q3d (one dose every three days). Semaglutide was synthesized by Eli Lilly and Company, Indianapolis, IN using protocols similar to published [40]. Compound was delivered just prior to dark cycle (0600–0700 hr) and continued for six doses. Dose volume was adjusted daily to maintain 10 ml/kg. Twenty-four hours following the last dose, animals were subjected to a 6 hour fast (0800–1400 hr) for performance of an oral glucose tolerance test (OGTT). Blood samples were obtained via tail clip at 0, 30, 60, 90 and 120 minutes post-glucose load (2 g/kg, PO) for assay of whole blood glucose. Animals were terminated by CO_2_ asphyxiation and cervical dislocation. Food consumption and body weight were recorded daily just prior to dark cycle (0600–0700 hr). The area of the blood glucose response curve corresponding to each animal was calculated by the trapezoid method [37], using each individual baseline blood glucose measurement prior to glucose administration as reference (t = 0). The sum of the trapezoidal areas between the 0, 30, 60, 90 and 120-minute time points corresponding to each animal were summed to obtain the area under the curve (AUC).

### Statistics

Except where mentioned, all data are presented as Mean ± SEM. Statistical analysis was done using Prism for Windows (version 6.07 GraphPad, San Diego, CA). As appropriate, one-way ANOVA or one-way ANOVA repeated measures followed by Dunnett’s multiple comparisons test were done. Also, two-way ANOVA and two-way repeated measures ANOVA followed by Sidak’s multiple comparison test were performed where different groups were studied over time.

## Results

### Age related changes in glucose homeostasis

**Fig 1:**
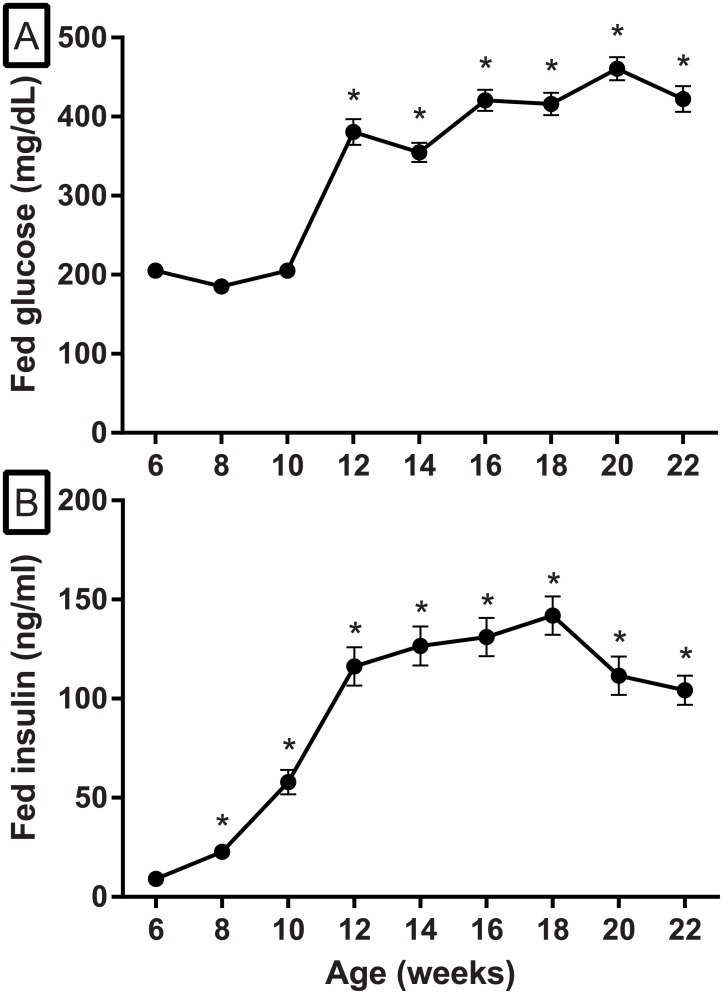
Post-prandial glucose (A) and insulin (B) concentrations in untreated male FATZO mice (6–22 weeks of age). Hyperglycemia developed spontaneously and was evident in animals as young as 6 weeks of age (A). Insulin responses to developing hyperglycemia create hyperinsulinemia during a period of marked insulin resistance (B). Each value represents the Mean ± SEM, n = 72. Analysis demonstrated increased glucose concentrations from baseline from 12–22 weeks of age and increases from baseline insulin concentrations from 8–22 weeks, (one-way repeated measures ANOVA, * *p* < .05 compared to baseline).

Serum glucose concentrations were determined in a cohort (n = 72) of FATZO mice every 2 weeks. Samples were obtained from conscious animals in the fed state. Hyperglycemia developed spontaneously and progressed quickly in FATZO mice when fed standard rodent chow. Serum glucose remained steady from 6–10 weeks of age (≈210 mg/dL), before a rapid increase to 380.4 ± 16.6 mg/dl was noted at 12 weeks. Glucose concentrations then increased more slowly and plateaued at ≅ 420 mg/dL as animals aged to 22 weeks (Fig 1A). Serum insulin concentrations were significantly higher in 6 week old FATZO animals compared to literature values for normal mice (9.15 ± 1.5 ng/ml vs. ≅ 0.5 to 1.5 ng/ml) (Fig 1B). Concurrent with the progressively increasing glucose concentrations, insulin concentrations rose six fold to 57.9 ± 6.1 ng/ml in 10 week old animals and fifteen fold to 142.0 ± 9.7 ng/ml in 18 week old animals. When glucose concentrations were in the mid-400 mg/dL range after 18 weeks of age, insulin concentrations began to fall (Fig 1A and 1B).

### Comparison to age-matched control mice

A small cohort of FATZO mice was compared to control C57BL/6J mice (n = 6 each) from 6–18 weeks of age. Compared to control mice, FATZO mice develop hyperglycemia, hyperinsulinemia and hypertriglyceridemia spontaneously when fed a standard rodent chow. Insulin resistance, as shown by abnormal glucose disposal, was apparent as early as 6 weeks of age and was concurrent with the accumulation of excess whole body fat.

**Fig 2:**
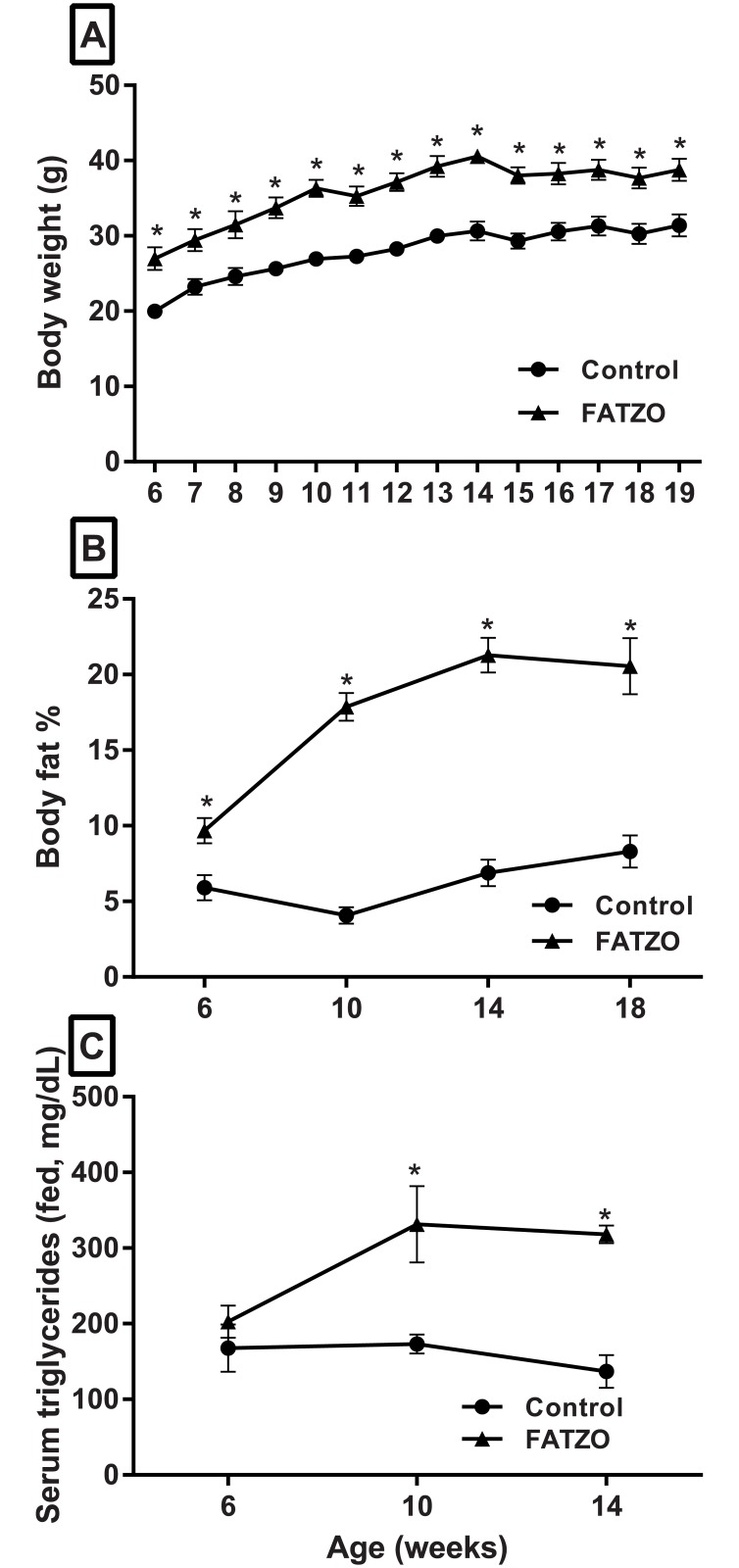
Body weight, body fat and serum triglycerides in untreated male FATZO mice compared to control mice. FATZO mice (▲) were significantly heavier (A) compared to age-matched C57BL/6J control mice (●) at each age (Mean ± SD). Increased levels of body fat contributes to increased body weight in FATZO mice (B). Body fat in FATZO mice (▲) was significantly higher when compared to control mice (●) from 6–18 weeks of age. Post-prandial serum triglycerides increased in untreated male FATZO mice (▲) as they aged and were significantly elevated when compared to control (●) mice at 10 and 14 weeks of age (n = 6, two-way repeated measures (A, B) or two-way ordinary ANOVA (C), * *p* < .05 when compared to control).

When fed a standard rodent chow, FATZO mice were significantly heavier compared to age matched control mice throughout the study. Six-week-old FATZO mice weighed 27.0 ± 0.6 g at baseline and gained weight steadily, reaching 38.8 ± 0.6 g at 19 weeks of age. Six-week-old control mice weighed 20.0 ± 0.6 g and grew to 31.4 ± 0.6 g by study end (Fig 2A). The body composition of FATZO mice was also significantly different compared to control mice from 6–18 weeks of age. A higher percentage of body fat was noted in 6 week old FATZO mice compared to control mice (9.7 ± 0.9 vs. 5.9 ± 0.8%, *p* < .05) and at 18 weeks (20.6 ± 1.8 vs. 8.3 ± 1.0%, *p* < .05) (Fig 2B). Feed intake (cumulative) over the course of the study was not significantly different between these groups of animals (310.0 ± 23.0 g for C57BL/6J vs. 330.0 ± 5.6 for FATZO). Serum triglycerides (fed) remained steady in control mice from 6–14 weeks of age, ranging from 167.8 ± 31.1 to 137.0 ± 21.9 mg/dL. Serum triglycerides in FATZO mice were slightly higher although not significantly different compared to control mice at 6 weeks of age (202.6 ± 21.2 vs. 167.8 ± 31.4 mg/dL). Triglycerides were significantly higher in FATZO mice at 10 weeks (331.3 ± 50.6 vs. 173.3 ± 12.9 mg/dL, *p* < .05) and 14 weeks of age (318.2 ± 11.6 vs. 137.0 ± 21.9 mg/dL, *p* < .05) (Fig 2C).

**Fig 3:**
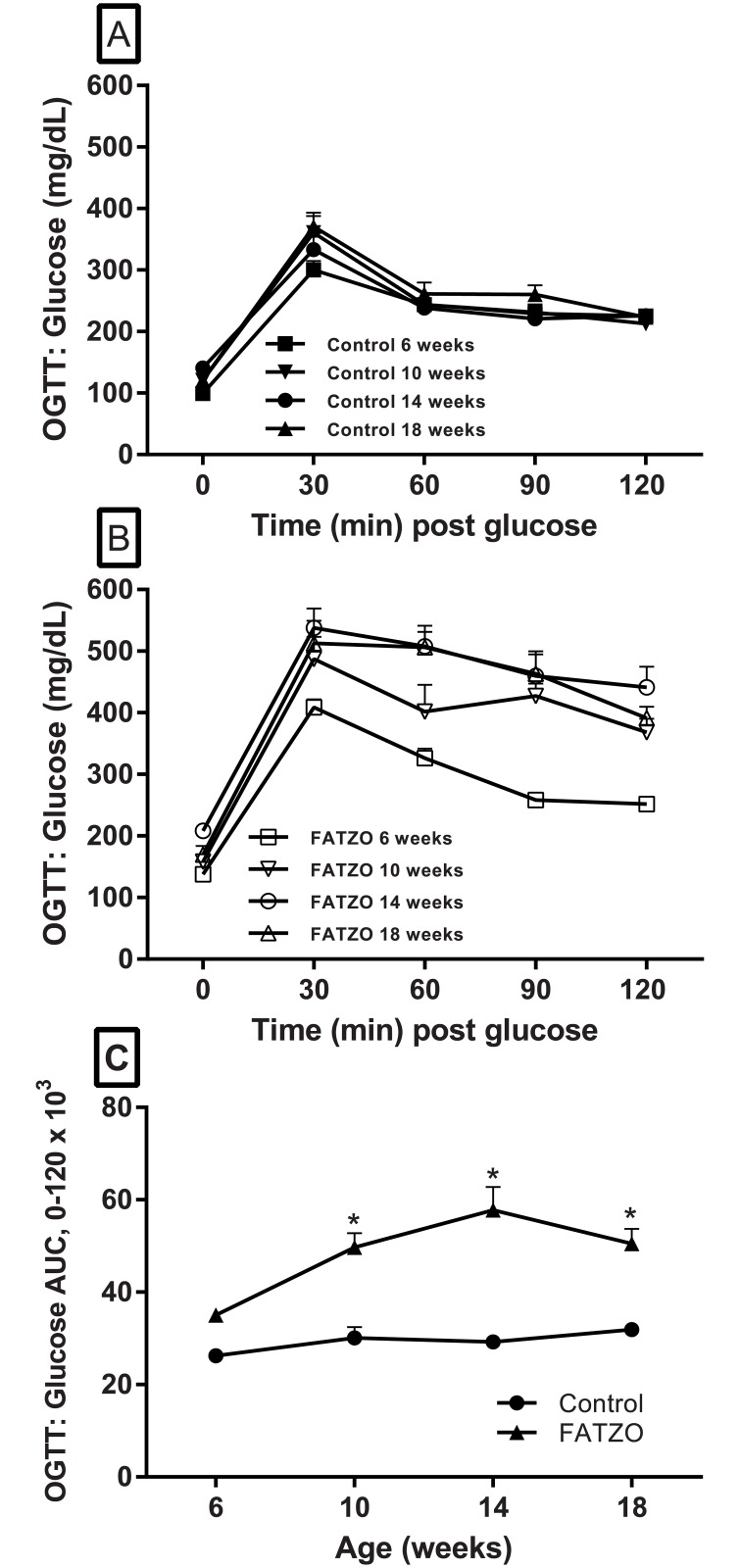
Glucose responses to a glucose load during performance of oral glucose tolerance test (OGTT) in control (A), FATZO mice (B) and the areas under the curve for both groups (C). An age-dependent impairment in glucose handling was apparent in FATZO mice compared to control mice in mice as young as 6 weeks. Glucose AUC (C) increased with age in FATZO mice (▲) compared to control mice (●) (two-way repeated measures, ANOVA * *p* < .05 when compared to control).

Oral glucose tolerance tests (OGTT) performed monthly on control mice indicated relatively stable disposal of the glucose load from 6–18 weeks of age (Fig 3A). In contrast, abnormal glucose disposal was prominent in FATZO mice as early as 6 weeks of age. The ability to handle the glucose load deteriorated with age in FATZO mice (Fig 3B). When represented as the area under the curve, the glucose AUC following a glucose load was not significantly higher in FATZO mice at 6 weeks but did reach significance when compared to control mice at 10, 14 and 18 weeks (Fig 3C).

### Effect of rosiglitazone and metformin

In a parallel study, we examined the effect of, rosiglitazone and metformin, on metabolic disturbances in the FATZO mouse model of type 2 diabetes/metabolic syndrome.

Body weight in ten-week old FATZO mice averaged 36.0 ± 0.4 g at study start. Administration of rosiglitazone 10 mg/kg/day elicited a significant increase in body weight compared to vehicle treated animals over the 8-week observation period (1.8 ± 0.2 vs. 5.3 ± 0.7 g, p < .05). In contrast, metformin treatment resulted in a slight although significant loss of body weight compared to vehicle treated animals (1.8 ± 0.2 vs. -0.2 ± 0.6 g, *p* < .05).

Serum glucose concentrations in the fed state were 315.6 ± 35.4 mg/dL in animals administered vehicle for 8 weeks. Serum glucose concentrations were somewhat lower compared to vehicle following treatment with rosiglitazone but did not reach statistical significance (315.6 ± 35.4 vs. 233.3 ± 7.7 mg/dL) and significantly reduced with metformin (315.6 ± 35.4 vs. 250.0 ± 12.8 mg/dL, p < .05).

**Fig 4:**
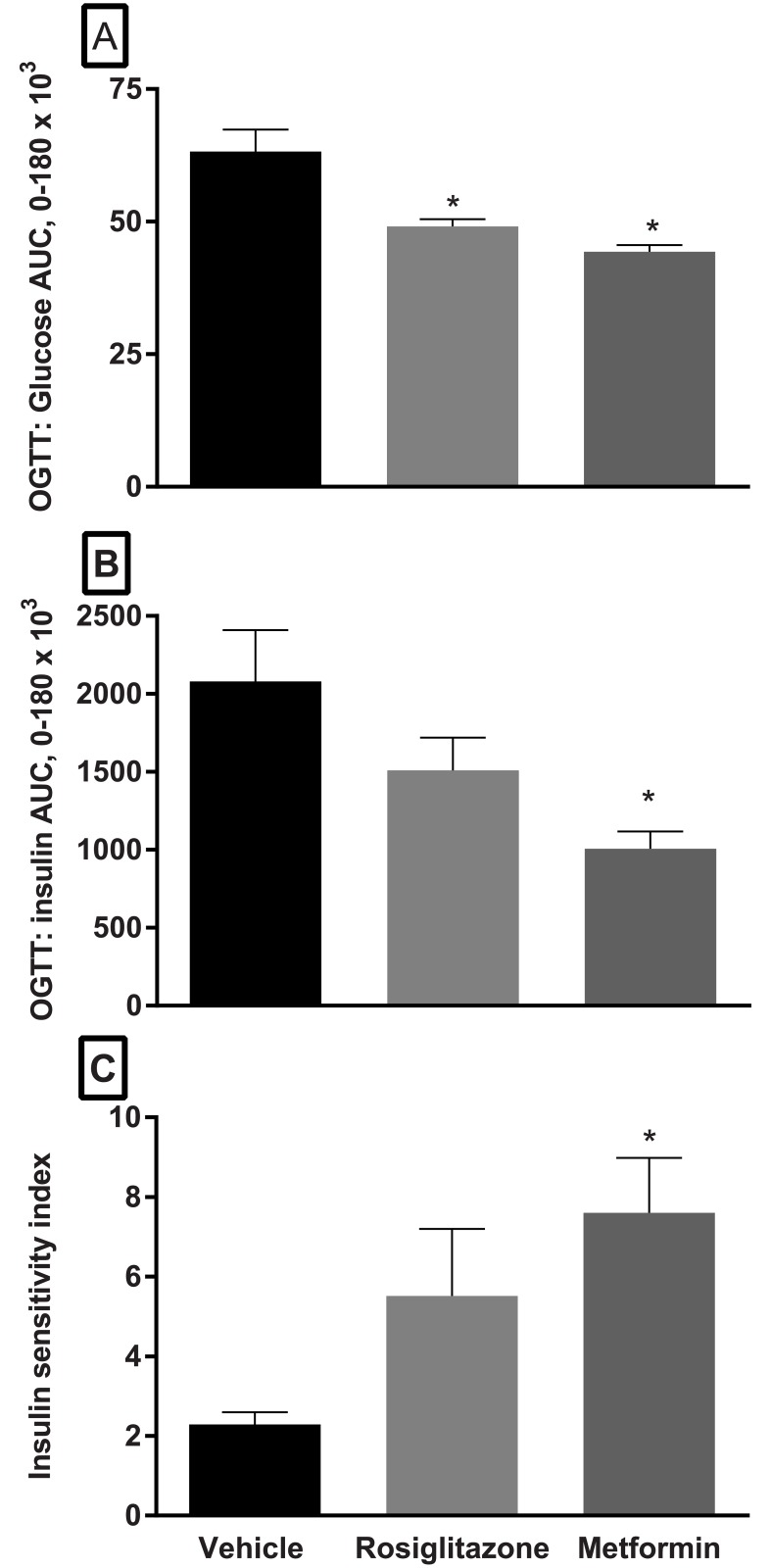
Area under the curve (AUC) analysis of glucose (A) and insulin (B) responses during OGTT and calculated insulin sensitivity index (C)(ISI) in FATZO mice following an 8-week administration of rosiglitazone or metformin. Both insulin sensitizers elicited significant reductions in the AUC for glucose in FATZO mice (A). Although it is reduced, insulin AUC did not reach significance for the rosiglitazone group but it did for the metformin group (B). Significant improvement in ISI was also demonstrated with metformin treatment and, although improved, rosiglitazone treatment did not reach statistical significance when compared to vehicle (one-way ANOVA, * *p* < .05 when compared to vehicle).

The area under the curve (AUC) for glucose (Fig 4A) was reduced significantly compared to vehicle following administration of both compounds for 8 weeks. However, only metformin elicited significant improvements in insulin AUC and in the calculated insulin sensitivity index (ISI) (Fig 4B and 4C).

### Effect of the GLP-1 receptor agonist semaglutide

**Fig 5:**
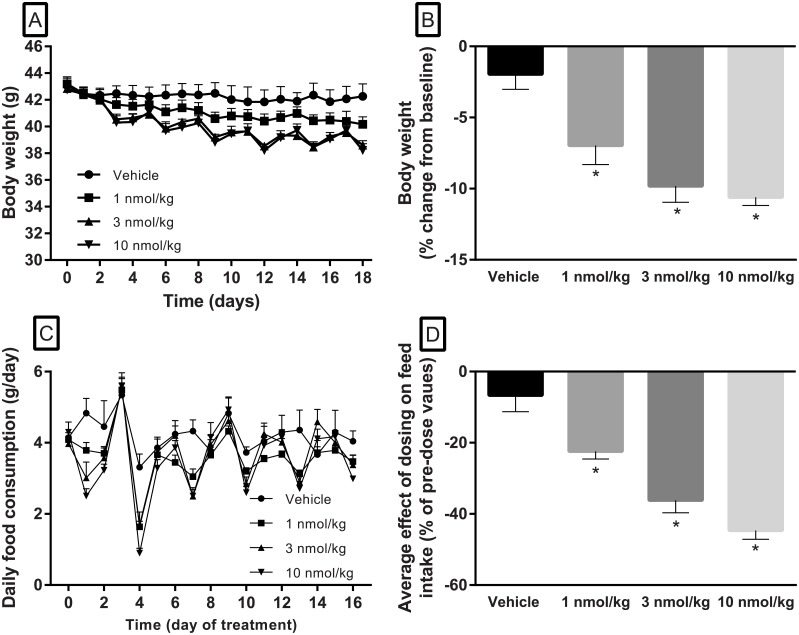
Body weight food intake in male FATZO mice during administration of semaglutide (1–10 nmol/kg, SQ, q3d, x 16 days). Semaglutide elicited dose-dependent decreases in body weight compared to vehicle within 2 days of start of treatment (A). At study end, animals administered semaglutide lost significantly more body weight compared to baseline values than vehicle treated animals (B). Daily variation in feed intake was apparent in all groups (C). A transient, dose-dependent reduction in feed intake compared to pre-dose values was observed during the 24 hrs following each semaglutide administration (D). Of note, a boiler failure resulted in decreased humidity of about 25% for one day which correlated with a transient increase in feed intake between day 2 and 3 [Vehicle ●, 1 nmol/kg ■, 3 nmol/kg ▲, and 10 nmol/kg ▼] (one-way ANOVA, * *p* < .05 when compared to vehicle).

Body weight in FATZO mice at baseline (15–16 weeks of age) averaged 43.0 ± 0.2 g and there was no difference among treatment groups at baseline (43.1 ± 0.5, 43.2 ± 0.5, 42.9 ± 0.5 and 42.7 ± 0.5 g for vehicle, and semaglutide at 1, 3 and 10 nmol/kg, respectively). Body weight in vehicle treated animals remained relatively steady throughout the study, losing 1.9 ± 1.1% of body weight compared to baseline following 16 days of vehicle administration. In contrast, dose-dependent and progressive loss of body weight was noted in semaglutide treated animals. Over the course of the study, a dose-dependent reduction in body weight compared to baseline was observed following semaglutide at 1, 3 and 10 nmol/kg (7.0 ± 1.3, 9.9 ± 1.4 and 10.6 ± 0.6% for the 1, 3 and 10 nmol/kg dose, respectively). This weight loss was significant compared to vehicle for all doses administered (Fig 5A and 5B).

Baseline feed intake averaged 4.1 ± 0.09 g/day for the 6 days prior to study start. Food consumption in semaglutide treated animals was significantly reduced compared to vehicle for the 24 hours following each drug administration (day 1, 4, 7, 10, 13, and 16). Averaged over the six drug administrations, the effect of semaglutide on food consumption (% reduction in food intake compared to pre-dose values) was significantly greater when compared to vehicle (-6.7 ± 4.6%) in all semaglutide groups (-22.4 ± 2.2, -36.1 ± 3.5 and -44.7 ± 2.5% for semaglutide at 1, 3 and 10 nmol/kg, respectively). These effects were transient as food consumption recovered to pre-dose values prior to next dose (Fig 5C and 5D).

**Fig 6:**
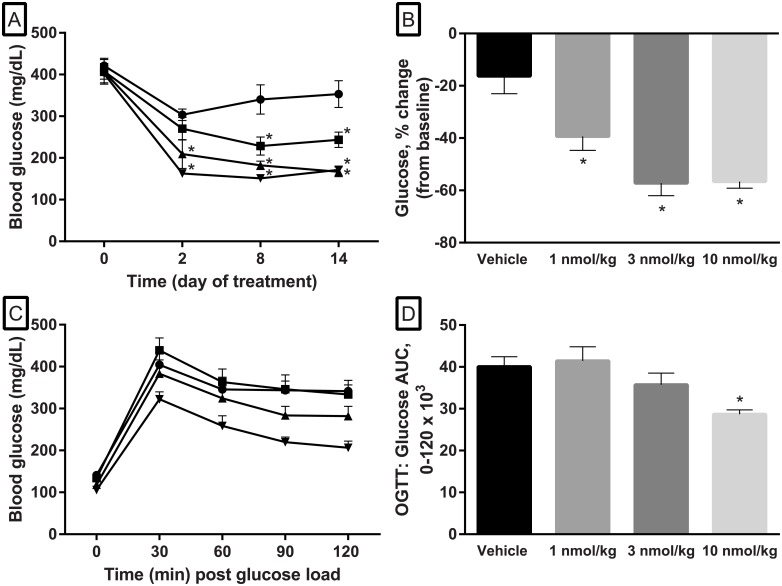
Changes in blood glucose and glucose responses to a glucose load during performance of oral glucose tolerance test in male FATZO mice following administration of semaglutide. Post-prandial glucose measured 24 hours after administration of semaglutide was dose-dependently reduced compared to vehicle over the course of the study (A) (two-way ANOVA, * *p* < .05 when compared to baseline). Terminal glucose data were also plotted as % decrease in glucose concentrations compared to baseline. The responses of all of the semaglutide groups were significantly higher than that of vehicle treated animals (B). Improvements in glucose handling were dose-dependent and significant compared to vehicle when administered at 10 nmol/kg (C, D) [Vehicle ●, 1 nmol/kg ■, 3 nmol/kg ▲, and 10 nmol/kg ▼] (one-way ANOVA, * *p* < .05 when compared to vehicle).

Baseline blood glucose averaged 408.9 ± 11.0 mg/dL in animals selected for study. Fed blood glucose measured twenty-four hours after the first, third and fifth dose was significantly reduced in semaglutide treated animals compared to those administered vehicle except for the 1 nmol/kg group after the first dose. Following the fifth administration, glucose values were reduced compared to baseline by 16.3 ± 6.8, 39.4 ± 5.3, 57.3 ± 4.8 and 56.6 ± 2.5% for vehicle, and semaglutide at 1, 3 and 10 nmol/kg, respectively (Fig 6A and 6B).

Administration of semaglutide elicited an improvement in glucose disposal. A dose-dependent reduction in the glucose AUC following an oral glucose tolerance test (OGTT) was observed following administration of semaglutide (Fig 6A and 6B). A reduction compared to vehicle (-11%) was observed following administration of semaglutide (3.0 nmol/kg); however, this response did not reach significance. The reduction in AUC was significant compared to vehicle for the 10 nmol/kg dose (40.04 ± 2.4, 41.5 ± 3.4, 35.7 ± 2.8 and 28.7 ± 1.0 AUC for vehicle and semaglutide at 1, 3 and 10 nmol/kg, respectively) (Fig 6C and 6D).

## Discussion

Metabolic syndrome is a multi-faceted condition stemming from dysregulation in multiple biochemical pathways. Patients often present with obesity, hypertension, dyslipidemia and abnormal glucose handling. While lifestyle changes to reduce obesity can add significant benefit, the cluster of these components often necessitate a pharmacological approach. Although metabolic syndrome likely represents concurrent signaling disruptions from several genes, the reason why several cardiovascular risk factors cluster in some individuals is not known. Since this is a likely hypothesis, pre-clinical screening of potential treatment regimens might therefore optimally be carried out in a polygenic rodent model with disease that closely mimics the human condition [41].

Three mouse models are being used currently in a majority of studies in obesity and diabetes; *db/db*, *ob/ob* and DIO mice primarily on a C57BL/6 background. The *db/db* and *ob/ob* mice have leptin receptor and leptin defects respectively while the DIO mice require high fat diets to become obese. The leptin receptor and leptin defects result in an obese phenotype that leads to insulin resistance, glucose resistance and hyperglycemia. However, defects in the leptin pathway are extremely rare in the human population [16–20]. They also interfere with normal hypothalamic signaling pathways, which compromises the testing of compounds that have CNS activity [42,43]. DIO mice require high fat diets to become insulin resistant and glucose intolerant; however, these models develop only moderate hyperglycemia. Although a number of alternate models to *db*/*db*, *ob*/*ob* and DIO have been developed such as TALLYHO/JngJ and NONcNZO10Lt/J [44,45]. According to a review of these models [44] they are remarkably similar with polygenic obesity, maturity onset diabetes and moderate leptin resistance. FATZO has all of these characteristics but appears to be more obese, has very high insulin and leptin levels and becomes leptin resistant as they age [46]. Although all these polygenic models have very interesting characteristics none have achieved the acceptance of *db*/*db*, *ob*/*ob* and DIO models. Since human obesity is rarely associated with impaired leptin signaling through defects in this pathway [15–19], the absence of a functional leptin pathway in the aforementioned models may hinder translation of pre-clinical results. The FATZO mouse was developed to increase the predictive impact of pre-clinical research in this area. Data presented here on the phenotype and response to current therapies support the contention that the FATZO mouse exhibits disease characteristics which mimic the human condition. As such, it may represent a reliable alternative for evaluation of agents directed towards improvement in significant health issues related to obesity, metabolic syndrome, type 2 diabetes and complications of these conditions.

This model has been under development for a number of years, and initially was bred by crossing C57BL/6J and AKR/J mice. Animals from this cross demonstrated high weights with significantly elevated glucose and insulin concentrations. Based on these data, a program of selective inbreeding was initiated to take the model to genetic homogeneity (30+ generations). This has resulted in a model that exhibits obesity, metabolic syndrome and significantly elevated glucose concentrations. Most of the data presented in this first characterization of the FATZO model were produced using animals selected for study based on age alone; however, a body weight inclusion criterion was used in the evaluation of the effects of semaglutide.

Although the FATZO mouse becomes obese, has significant insulin resistance, hyperlipidemia and hyperglycemia, under the conditions of these experiments it does not develop severe hyperglycemia with low concentrations of insulin that would indicate beta cell loss. As a result, one could argue that, under these conditions, this is a model of metabolic syndrome or pre-diabetes. Human pre-diabetes is defined somewhat differently by different organizations [47]. The pre-diabetic condition can be modulated significantly by various factors [48–50]. Furthermore, the characteristic that is used most often to initially screen for pre-diabetes is impaired fasting glucose. This is obviously associated with other abnormalities that can be measured such as impaired glucose tolerance, increased HbA1c, HOMA-IR and other test results.

Although the conditions in animals are similar, the accurate translation of specific measurements from animals to humans is inherently difficult as size, metabolic rates and physiology are significantly different. Nevertheless, the results presented in this paper demonstrate the characteristics of the FATZO model as well as the response of the model to three compound classes that are effective in people.

Metformin is accepted as a first line therapy in patients with type 2 diabetes and is the cornerstone of oral blood glucose lowering therapy [51]. Metformin lowers fasting glucose and improves glucose tolerance in pre-diabetic [52,53] as well as overtly diabetic individuals [54]. Metformin effectively improves glucose disposal in high fat fed mice [55]. As monotherapy in humans, a modest loss of body weight occurs in non-diabetic [56–58] as well as obese diabetic patients [52,59]. In addition, administration of metformin is associated with weight loss in obese DIO mice [55,60,61].

Rosiglitazone is a thiazolidinedione (TZD) insulin sensitizer, which has been shown to favorably influence pancreatic beta cell survival and function in rodent models of diabetes [62,63]. Decreased insulin resistance and reduced hyperglycemia have been observed in humans [64,65] and in obese mice [66–68] following treatment with rosiglitazone. The major clinical side effect of rosiglitazone is significant weight gain [69,70]. Rosiglitazone is also associated with weight gain in diabetic obese mice [71,72] including the FATZO mice in this study.

The actions of glucagon-like peptide (GLP-1) on insulin release [73,74] and an apparent impairment in the hormones actions in diabetic patients [75] has led to the development of GLP-1 receptor agonists to improve glucose homeostasis in diabetic patients [76,77]. In patients whose diabetes is not adequately controlled by metformin or a TZD, GLP-1 receptor agonists are often added to reach treatment goals [78]. Ongoing efforts to maximize GLP-1 therapy have focused on lengthening the duration of action to decrease frequency of administration. Although semaglutide is a longer acting GLP-1 agonist and has demonstrated efficacy in humans when administered once weekly [79], it has a shortened period of effectiveness in rodents. Similar to other proteins such as gamma globulin [80] and albumin [81], internal data have demonstrated that the half-life of semaglutide is significantly shorter in rodents than in man. Based on these internal data we selected dosing every 3 days. Other long acting GLP-1 based receptor agonists are now clinically available [78,82]. The GLP-1 receptor agonists have also been shown to inhibit cumulative feed intake, reduce body weight and improve glucose tolerance in DIO mice [83–85].

Fast times were different in the studies based on wanting to compare some of the studies with previous work and a changing philosophy about the appropriate fasting time for rodents. Longer overnight fasts actually result in the fasting time plus the lower food intake in preceding daylight hours for rodents. A previously published paper from our laboratory successfully used 6-hour morning fasts [86]. Longer fasts could actually result in significant changes in glucose production [87]. Although we have not done side by side comparisons we feel that shorter fasts during a normally lower food consumption period, such as in typical human fasts, should more closely resemble the human situation.

The FATZO mouse represents a new mouse model with a phenotype similar to that seen in pre-diabetes and people with type 2 diabetes [88]. This mouse is heavier than age-matched control animals with a higher percentage of body fat. In addition, the mouse is hyperinsulinemic at an early age with impaired glucose disposal and elevated triglycerides when compared to normal mice. The obese, insulin resistant FATZO mouse responded to the three classes of anti-diabetic agents described above in a fashion comparable to that of humans and other obese models of type 2 diabetes [89–91]. Body weight reduction with improved glucose tolerance was observed in obese FATZO mice treated with metformin (150 mg/kg/day) and an improvement in glucose tolerance with significant weight gain followed rosiglitazone (10 mg/kg/day) treatment. Administration of semaglutide (1–10 nmol/kg, SQ, q3d) elicited a loss of body weight, improvement in glucose tolerance and an acute reduction in feed intake.

The FATZO mouse was developed for evaluating compounds to combat obesity, metabolic syndrome, diabetes. Currently prescribed anti-diabetic agents were effective in this model in a comparable fashion to their effectiveness in human type 2 diabetes. This model was designed to have an intact leptin pathway and demonstrates increasing leptin levels and less responsiveness to leptin as the animals age [46]. As a result, this model should prove effective in the evaluation of compounds that are designed to interact with CNS targets—particularly those focused on obesity mechanisms integrated within the hypothalamus and median eminence. Prolonged significant hyperglycemia in a model with functioning beta cells enables evaluation of agents designed to preserve beta cell function. Models such as the ZDF rat and the *db/db* mouse lose their beta cells very quickly once hyperglycemia develops leaving a very narrow window to look at mechanisms that center on beta cells and natural insulin release and function. We believe that the characteristics of the FATZO mouse model presented here validate it as a relevant model that can be used to study obesity, glucose intolerance, insulin resistance and type 2 diabetes and may be useful in the development of pharmaceuticals to treat these conditions.

Although the FATZO mouse was primarily developed to have an intact leptin pathway that did not require long-term feeding with high-fat diet to become obese and insulin resistant. Under these conditions, all of these are present in this model including a reasonably high hyperglycemic/pre-diabetic condition. If one uses this mouse without high-fat diet, there are significant price- and time-savings with a model that is much more hyperglycemic than the DIO mouse. If one does choose to feed these animals a DIO diet they become much more hyperglycemic and appear to develop full type 2 diabetes [46].
